# Clonal seborrheic keratosis: a rare skin tumor

**DOI:** 10.11604/pamj.2019.34.54.13415

**Published:** 2019-09-27

**Authors:** Marwa Bouhamed, Dhouha Bacha, Fadoua Abdelmoula, Sana Ben Slama, Ahlem Lahmar, Saadia Bouraoui, Mzabi-Regaya Sabeh

**Affiliations:** 1 Department of Pathology, Mongi Slim University Hospital, Tunis, Tunisia; 2 Department of Dermatology, Khaireddine Hospital, Tunis, Tunisia

**Keywords:** Clonal, keratosis, seborrheic

## Abstract

Seborrheic keratosis is a benign epidermal neoplasm, representing one of the most common skin tumors. Clonal seborrheic keratosis is one of the histological subtypes of this entity. It is an uncommon lesion which may resemble other benign or malignant lesion. We report a case of a 60-year-old woman presented with a 7 year history of a gradually growing, cutaneous lesion on her left arm. On physical examination, the lesion was elevated, well-circumscribed, measuring 5 cm in maximum diameter. The tumor was biopsied. Histopathological examination revealed the presence of well-defined nests of clear-looking or basaloid keratinocytes within an acanthotic epidermis, corresponding to the Borst-Jadassohn phenomenon. The tumor cells were small and monomorphic. We diagnosed this tumor as clonal seborrheic keratosis. Although surgical excision was recommended, our patient refused an operation. This case of clonal seborrheic keratosis is presented for its rarity and for differential diagnosis.

## Introduction

Seborrheic keratosis (SK) is one of the most common benign tumors of the skin. The clinical diagnosis of this entity is often simple. Clonal SK is an atypical variant which may resemble other benign or malignant lesion. We herein report a patient who clinically presented with reddish, focally dark-brown lesion that histopathologically showed a benign lesion mainly composed of clonal (“nested”) seborrheic keratosis. Through this report, we will highlight the clinical and histopathological features of this lesion and review the main differential diagnoses.

## Patient and observation

**Figure 1:**
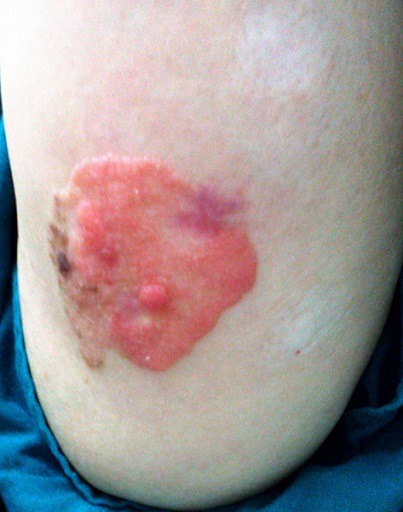
Elevated well-circumscribed lesion which is irregularly shaped, reddish, and focally dark-brown

**Figure 2:**
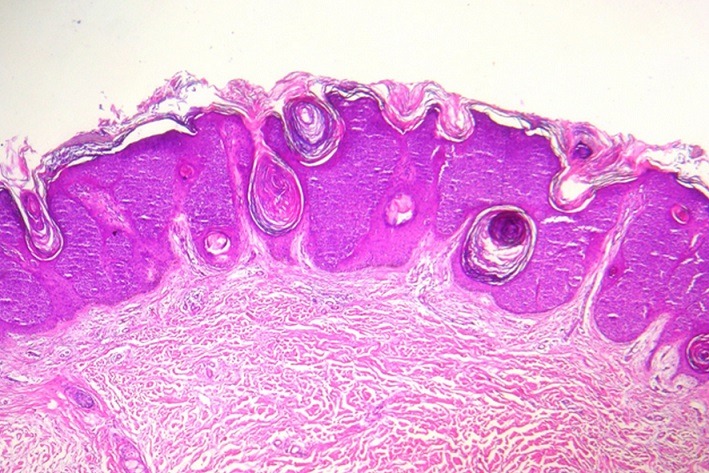
Well defined nests of clear-looking or basaloid keratinocytes within an acanthotic epidermis (hematoxylin eosin × 100)

**Figure 3:**
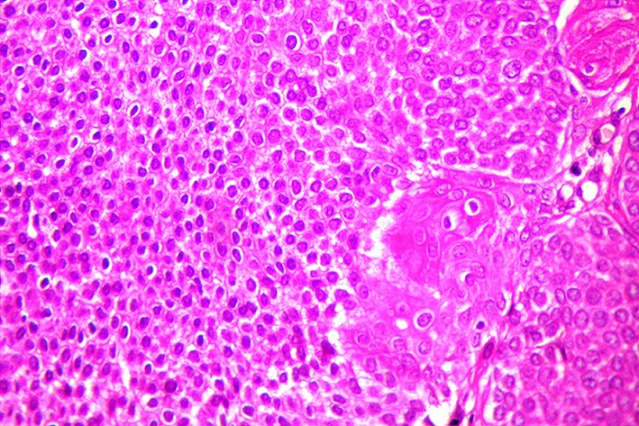
Artifactual retraction spaces surrounding the clusters of tumor cells (hematoxylin eosin × 400)

A 60-year-old woman presented with a 7 year history of a gradually growing, asymptomatic, cutaneous lesion on her left arm. Her medical and family history was unremarkable; especially there was no personal or family history of skin malignancy. On physical examination, the lesion was elevated, well-circumscribed, irregularly shaped, reddish, focally dark-brown, measuring 5 cm in maximum diameter (Figure 1). No regional lymph nodes were palpable. The patient did not feel any pruritus or pain at the lesion site. Dermoscopy was not performed. The tumor was biopsied. Histopathological examination revealed that the lesion had an irregular papillomatous surface with areas of hyperkeratosis, parakeratosis and acanthosis associated to pseudohorn cysts. Well defined nests of clear-looking or basaloid keratinocytes were present within the acanthotic epidermis, corresponding to the Borst-Jadassohn phenomenon (Figure 2). These clusters were surrounded by artifactual retraction spaces. The tumor cells were small and monomorphic (Figure 3). Cellular and nuclear atypical features were not present. The nuclei were round, with dense chromatin and numerous mitotic figures. The dermis displayed only mild chronic inflammation. We diagnosed this tumor as clonal seborrheic keratosis. Although surgical excision was recommended, our patient refused an operation.

## Discussion

Seborrheic keratosis is a benign epidermal neoplasm [1, 2]. It represents one of the more common skin tumors seen by dermatologists in clinical practice [1]. The etiology of SK is still unclear. Although, advanced age and ultraviolet irradiation may explain its development [2]. Clonal SK is one of the histological subtypes of this entity. It is an uncommon lesion, seen in adult and middle-aged patients [3]. It may be found anywhere on the body (excluding palms and soles) and it is especially common on the face, chest and back. Clinically, clonal SK cannot be differentiated from ordinary SK [4]. It appears as a sharply demarcated gray-brown to black lesion, which is slightly raised, covered sometimes with greasy scales. However, the clinical diagnosis of clonal SK may be challenging, miming both premalignant and malignant lesions. Dermoscopically, it reveals the presence of variously sized, blue-gray globular-like structures that are aggregated to form short lines or irregularly distributed within the lesion [5, 6]. It can reveal other features suggestive of SK, including demarcated borders, milia-like cysts, comedo-like openings, and the jelly sign [5, 6].

Dermoscopy does not reach 100% diagnostic accuracy and clonal SK may represent a dermoscopic trap, simulating either a melanocytic lesion or a basal cell carcinoma [1, 4, 5]. Histopathological examination should constantly be performed in cases in which dermoscopy exhibits confounding features that do not allow an accurate diagnosis [6]. Histologically, clonal SK is characterized by the presence of intraepithelial, well defined nests of basaloid or pale cells which correspond to the globular structures seen on dermoscopy examination [2, 5]. This intra-epidermal proliferation is known as Borst-Jadassohn phenomenon [1, 2]. In the histological differential diagnosis of clonal SK, we have to consider other benign tumor such as Hidroacanthoma Simplex (HS), epidermal nevus; and also malignant neoplasm such as Bowen’s disease and superficial Basal Cell Carcinoma. HS is a benign, uncommon skin tumor described as verrucous keratotic plaque clinically misdiagnosed as a seborrheic keratosis or in situ squamous cell carcinoma [3]. Histologically, HS is characterized by discrete intraepidermal circumscribed nests of basaloid and pale-staining cells within an irregularly acanthotic epidermis, miming clonal SK [3-5]. Therefore, it is difficult to differentiate it especially when the ductal structure and cystic space are absent [7]. In the literature, the differential diagnoses between HS and clonal SK was controversial because both tumors revealed very comparable patterns of cytokeratin expression [7, 8]. Currently, Takayama *et al.* reported that lumican is a powerful differential diagnostic marker that discriminates these two neoplasms [7, 9]. The author demonstrated that in 78.6% of HS cases, lumican was positive in poroid cells, whereas the tumor cells in most cases of clonal SK were negative [9]. Epidermal nevus may resemble clinically SK. Histologically; both tumors demonstrate hyperkeratosis, papillomatosis and acanthosis. In the clonal variant, both show intraepidermal, well demarcated nests of basaloid cells. In such cases, we can base on the history of the patient disease and the lesion geometry to lead one to the correct diagnosis. Epidermal nevi tend to have a linear configuration, whereas SK tends to be round to oval in shape and are often smaller. In this case, a clinicopathological confrontation is necessary.

Bowen’s disease affects elderly persons, like SK. It is characterized by acanthosis with entire disorganization of the epidermal architecture, lack of polarity of cells, and loss of maturation. The tumor cells are large with hyperchromatic atypical nuclei and abundant cytoplasm. Numerous mitotic figures, including atypical forms, may be observed. Superficial Basal Cell Carcinoma consists of superficial lobules of basaloid cells, which project from the epidermis or from the sides of follicles or eccrine ducts. This neoplastic basaloid lobules are not circumscribed within the epidermis and tend to project to the papillary dermis [5]. Like all subtypes of SK which are benign, the clonal variant can act as cutaneous markers for internal malignancy. Indeed, sudden onset of numerous SK (Leser-Trélat sign) has been reported in association with internal malignancy. The most common neoplasm reported with this sign is the adenocarcinoma of the stomach. The occurrence of SK in association with malignant skin tumors is uncommon. They arise within the tumor or related to it. In one retrospective case series, only 9% of 639 histologically diagnosed SKs were found to be associated with other lesions [10]. Of these associated lesions, only 44 cases were malignant [10]. However, in this article, the different histological subtypes of SK were not mentioned. The most common association was with basal cell carcinoma, squamous cell carcinoma, keratoacanthomas, Bowen disease and malignant melanomas. To our knowledge, there is no sufficient data in the literature to understand the association between SK and malignant lesions. Therefore, it may be an incidental phenomenon. If appropriate, SK could represent a precursor lesion. Because clonal SK is benign, treatment is not mandatory, but many patients seek treatment for removal of SK for cosmetic reasons. The therapy of choice is ablation of the lesion by curettage, shave excision, electrocautery, cryotherapy, or ablative laser systems. However, if SK appears to be atypical or inflamed, biopsy or removal is recommended.

## Conclusion

In conclusion, although clonal SK is a benign disorder, there are various indications for the removal of lesions especially when it has a suspicious clinical presentation.

## Competing interests

They authors declare no competing interests.
